# Effect of vitamin D on aortic remodeling in streptozotocin-induced diabetes

**DOI:** 10.1186/1475-2840-11-58

**Published:** 2012-07-13

**Authors:** Erik Salum, Priit Kampus, Mihkel Zilmer, Jaan Eha, Mark Butlin, Alberto P Avolio, Taavi Põdramägi, Andres Arend, Marina Aunapuu, Jaak Kals

**Affiliations:** 1 Department of Cardiology, University of Tartu, 8 Puusepa Street, Tartu, 51014, Estonia; 2 Endothelial Centre, University of Tartu, 8 Puusepa Street, Tartu, 51014, Estonia; 3 Department of Biochemistry, Centre of Excellence for Translational Medicine, University of Tartu, 19 Ravila Street, Tartu, 50411, Estonia; 4 The Australian School of Advanced Medicine, 2 Technology Place, Macquarie University, NSW, 2109, Australia; 5 Department of General and Molecular Pathology, University of Tartu, 19 Ravila Street, Tartu, 50411, Estonia; 6 Department of Anatomy, University of Tartu, 19 Ravila Street, Tartu, 50411, Estonia; 7 Department of Vascular Surgery, Tartu University Hospital, 8 Puusepa Street, Tartu, 51014, Estonia

**Keywords:** Streptozotocin, Aortic stiffness, Pulse wave velocity, Elastin, Asymmetric dimethylarginine, Vitamin D

## Abstract

**Background:**

Diabetes mellitus is associated with micro- and macrovascular complications and increased cardiovascular risk. Elevated levels of serum asymmetric dimethylarginine (ADMA) may be responsible for endothelial dysfunction associated with diabetes-induced vascular impairment. Vitamin D may have potential protective effects against arterial stiffening. This study aimed to examine both the effects of diabetes on the functional/structural properties of the aorta and the endothelial function and the effects of vitamin D supplementation.

**Methods:**

Male Wistar rats (n = 30) were randomly assigned to control untreated, diabetic untreated, and diabetic + cholecalciferol groups. Diabetes was induced by intraperitoneal injection of streptozotocin, followed by oral administration of cholecalciferol (500 IU/kg) for 10 weeks in the treatment group. Aortic pulse wave velocity (PWV) was recorded over a mean arterial pressure (MAP) range of 50 to 200 mmHg using a dual pressure sensor catheter. Intravenous infusion of phenylephrine and nitroglycerine was used to increase and decrease MAP, respectively. Serum 25-hydroxyvitamin D [25(OH)D] levels were measured using a radioimmune assay. ADMA levels in serum were measured by enzyme-linked immunoassay. Aortic samples were collected for histomorphometrical analysis.

**Results:**

PWV up to MAP 170 mmHg did not reveal any significant differences between all groups, but in diabetic rats, PWV was significantly elevated across MAP range between 170 and 200 mmHg. Isobaric PWV was similar between the treated and untreated diabetic groups, despite significant differences in the levels of serum 25(OH)D (493 ± 125 nmol/L *vs* 108 ± 38 nmol/L, respectively). Serum levels of ADMA were similarly increased in the treated and untreated diabetic groups, compared to the control group. The concentration and integrity of the elastic lamellae in the medial layer of the aorta was impaired in untreated diabetic rats and improved by vitamin D supplementation.

**Conclusion:**

PWV profile determined under isobaric conditions demonstrated differential effects of uncontrolled diabetes on aortic stiffness. Diabetes was also associated with elevated serum levels of ADMA. Vitamin D supplementation did not improve the functional indices of aortic stiffness or endothelial function, but prevented the fragmentation of elastic fibers in the aortic media.

## Background

Cardiovascular (CV) events are considered to be a principal cause of mortality in patients with diabetes mellitus (DM) [1]. Macrovascular complications of DM are associated with stiffening of the aorta, which is a major contributing factor to the target organ damage, such as impaired coronary perfusion [2] and cardiac hypertrophy [3]. Aortic stiffness can be assessed with measurement of aortic pulse wave velocity (PWV) that is largely determined by the structural properties of the vessel wall and blood pressure (BP) level. PWV is regarded as a strong and independent predictor of CV complications in patients with DM [4].

Asymmetric dimethylarginine (ADMA) is an endogenous inhibitor of nitric oxide synthase [5] which is produced by vascular endothelial cells [6]. Elevated levels of ADMA in plasma have been found associated with impaired endothelial function [7] and decreased arterial elasticity [8], characterised by decreased bioavailability of nitric oxide (NO). There is substantial evidence that endothelial dysfunction in diabetes is directly associated with increased aortic stiffness [9].

It is now widely recognised that vitamin D not only plays a major role in bone and calcium metabolism, but may also improve CV health and reduce the risk of CV morbidity and mortality [10,11]. There is evidence that hypovitaminosis D may adversely affect endothelial function [12], leading to increased aortic stiffness [13]. The association between vitamin D deficiency and increased aortic stiffness has been demonstrated in healthy subjects [14,15] and in different chronic inflammatory diseases, including systemic lupus erythematosus [16] and DM [17,18]. Furthermore, the administration of vitamin D to subjects with DM been shown to improve insulin secretion and insulin resistance [19]. However, the potential role of vitamin D in diabetic macrovascular complications remains unclear. We designed the current study to investigate the effects of streptozotocin-induced diabetes on the functional/structural properties of the aorta and endothelial function and the potential protective effects of vitamin D supplementation.

## Materials and methods

### Animals

The experiments were performed in 30 male Wistar rats (RccHan:WIST, age 4 months) obtained from Harlan Laboratories (Harlan Laboratories, Inc., The Netherlands). The animals were kept in a room with controlled temperature (21 ± 2°C) and lighting (12:12-h light–dark cycle) with free access to food pellets and tap water. All experimental procedures were approved by the Estonian National Board of Animal Experiments and were conducted in accordance with the European Communities Directive (86/609/EEC).

### Treatment

Rats were randomly assigned to three groups of equal size: control group, diabetic group, and cholecalciferol-treated diabetic group. Diabetes was induced by a single intraperitoneal injection of streptozotocin (STZ) 50 mg/kg (Sigma-Aldrich, St. Louis, MO, USA) freshly dissolved in 0.9% NaCl solution. Blood samples were taken 48 h later from the tail vein and glucose levels were measured with a glucometer (Glucocard X-meter, Arkray Inc., Japan). Rats with glucose levels >15 mmol/L were considered diabetic. One animal died 2 days following STZ injection and another animal did not develop hyperglycemia. Immediately after confirmation of diabetes, one diabetic group of animals was submitted to supplementation with cholecalciferol (Sigma-Aldrich, St. Louis, MO, USA) 12.5 μg (500 IU) kg^-1^ body weight, dissolved in 0.3 ml olive oil administered orally. This dose was expected to be below that which causes hypercalcemia and soft tissue calcification since animal models of arterial wall calcification require the administration of much higher doses of vitamin D [20]. Cholecalciferol was chosen over calcitriol, the hormonal form of vitamin D, to further reduce the risk of soft tissue calcification. Cholecalciferol was administered every other day by gavage for a period of 10 weeks. Weekly, body weight was monitored and glycosuria was assessed with reagent strips (Combur Test, Roche, Germany) to exclude ketosis.

### Haemodynamic measurements

After 10 weeks, the animals were anesthetised with a mixture of fentanyl (0.07 mg/kg, Gedeon-Richter Plc., Hungary), midazolam (5 mg/kg, Roche Pharma AG, Germany), and ketamine (75 mg/kg Vetoquinol Biowet Sp. z.o.o., Poland) administered subcutaneously. The optimal concentrations of the anesthetic substances were determined in pilot experiments. After induction of anesthesia, animals were placed on a heating pad and body temperature was maintained at 37°C. A 2.5 F high-fidelity, dual pressure sensor catheter with 50 mm separation between sensors (SPC-721, Millar Instruments Inc., TX, USA) was introduced via the femoral artery into the descending aortic trunk so that the distal sensor was positioned at the beginning of the descending aorta and the resulting position of the proximal sensor was just proximal to the aortic bifurcation. Mean arterial pressure (MAP) was determined from measurements made by the proximal pressure transducer. Arterial pressure was increased and decreased by infusion of phenylephrine (50 μg/min) and nitroglycerine (30 μg/min), respectively, via a catheter inserted into the femoral vein. Measurements of PWV were performed similarly to what has been described by other investigators [21,22]. Briefly, pulse pressure waves were recorded simultaneously at the two aortic sites and PWV was calculated by dividing the propagation distance by propagation time using an automated foot-to-foot method. The foot of the pressure wave was defined by the peak of the second time derivative of pressure during each pulse. As the distance between the sensors is fixed at 50 mm, this calculation provides a highly accurate measurement of PWV. Data were acquired at a sampling rate of 2 kHz (PowerLab, ADInstruments, Australia) and feature extraction and calculations made with custom scripts in Spike2 v.6. software (Cambridge Electronic Design, UK). PWV was plotted against MAP at 5 mmHg increments to characterise PWV over a wide range of MAP from 50 to 200 mmHg.

### Laboratory parameters

After the haemodynamic measurements were completed, blood samples were taken from the tail vein for assessment of glucose levels. The rats were euthanised by drawing blood by cardiac puncture; part was used to measure glycated haemoglobin (HbA1c) level and the remaining portion was centrifuged at 3000 rpm for 15 minutes to obtain serum. Serum 25-hydroxyvitamin D [25(OH)D] level was measured using a radioimmune assay (25-Hydroxyvitamin D, ^125^I Ria Kit, Diasorin Corporation, USA). ADMA was determined from serum samples by an enzyme-linked immunoassay using a commercial kit (DLD Diagnostika, Germany). Calcium concentration in serum was determined by a colorimetric test (Calcium liquicolor, HUMAN Gesellschaft für Biochemica und Diagnostica mbH, Germany).

### Histological analysis and morphometric parameters

The aortic samples for histological analysis were fixed in 10% formalin for 12 hours and embedded in paraffin with vacuum infiltration processor (Tissue-Tek® VIP^TM^ 5 Jr, Sakura, USA). Specimens were cut with microtome Ergostar HM 200 (Microm, Germany) at four-μm thickness sections and stained using the hematoxylin-eosin, resorcin-fuchsin, and van Gieson methods for examination by light microscopy (Olympus BX50, Japan).

Estimation of the internal diameter of the aorta was performed by measuring two inner diameters at right angle for each cross-section of the thoracic aorta. At least eight different cross-sections of the aorta were analysed for each rat.

Thickness of the medial layer of the aorta was determined in thoracic aorta cross-sections by ten consecutive measurements in a systematic manner to evaluate all segments of the circumference of the aorta. At least six different cross-sections of aorta were analysed for each rat.

The staining intensities of the elastic fibers in the media and collagen fibers in the media and adventitia were evaluated on a subjective scale ranging from 0 to 3 (0 – no staining of fibers, 1 – poor staining of fibers, 2 – moderate staining of fibers, 3 – intensive staining of fibers). The evaluations were performed by two independent observers in a blinded fashion; the scores were summed and used for statistical analysis.

### Statistical analysis

Results are expressed as means ± standard deviation (SD). Differences between the groups were evaluated using the one-way analysis of variance (ANOVA) followed by Tukey’s *post-hoc* analysis for multiple comparisons of group means. Semi-quantitative data were compared by the Kruskal-Wallis one-way ANOVA followed by Mann–Whitney *U* test. Differences were considered to be statistically significant when *p* was <0.05. All statistical comparisons were performed with Statistica software (version 8; StatSoft, USA).

## Results

### Basic and biochemical parameters

The results are presented in Table 1. The initial body weights were similar in control and diabetic groups. The final body weights in the diabetic groups were significantly lower than in the control group. There were no differences between the diabetic treated and untreated groups in body weight after the treatment period. The levels of blood glucose and HbA1c were found to be significantly increased in the diabetic rats and were not affected by the vitamin D supplementation. Serum 25(OH)D level was significantly decreased in the untreated diabetic group, compared to the control group. Administration of vitamin D resulted in a significantly higher serum 25(OH)D level than that in the control group. Serum levels of ADMA were significantly lower in the control group compared with both diabetic groups. Vitamin D supplementation did not prevent the elevation of serum ADMA concentration. Serum calcium levels were similar between all groups, indicating that the administered dose of cholecalciferol remained within safe limits without increasing the risk of soft tissue calcification and possibly contributing to aortic stiffening.

**Table 1:** Basic and laboratory parameters

Group	Body weight (g)		Blood glucose (mmol/L)	HbA1c (%)	25(OH)D (nmol/L)	ADMA (μmol/L)	Calcium (mmol/L)
	Before	After					
Control (n = 10)	405 ± 26	450 ± 30	6.3 ± 1.6	4.0 ± 0.1	140 ± 21^#^	0.68 ± 0.18	2.7 ± 0.3
Diabetes (n = 9)	406 ± 56	370 ± 50^*^	28.3 ± 3.9^*^	10.3 ± 0.7^*^	108 ± 38^#¶^	0.87 ± 0.14^¶^	2.6 ± 0.3
Diabetes + vitamin D (n = 10)	406 ± 51	352 ± 43^*^	28.5 ± 5.9^*^	9.5 ± 1.3^*^	494 ± 125	0.85 ± 0.16^¶^	2.7 ± 0.2

Body weight was assessed at the beginning and at the end of the experiment.

HbA1c, glycated haemoglobin; ADMA, asymmetric dimethylarginine; 25(OH)D, 25-hydroxyvitamin D.

^¶^*p* < 0.05 vs control; ^*^p < 0.001 vs control; ^#^*p* < 0.001 vs diabetes + vitamin D.

### Haemodynamic parameters

**Figure 1:**
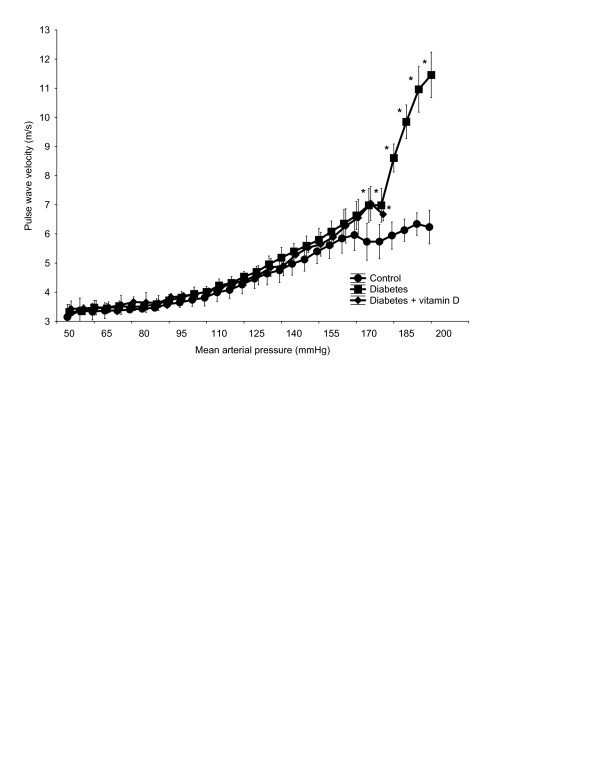
**Isobaric PWV-MAP curves in the control (n = 10), untreated diabetic (n = 9), and diabetes + vitamin D (n = 9) groups, averaged over 5 mmHg pressure steps.** * *p* < 0.05 vs control.

Before administration of the vasoactive substances, resting systolic blood pressure (SBP), diastolic blood pressure (DBP), pulse pressure (PP), mean arterial pressure (MAP), and heart rate (HR) were not statistically different between all groups (Table 2). Intravenous infusion of phenylephrine increased MAP to 200 mmHg, followed by infusion of nitroglycerine, which decreased MAP to 50 mmHg. The diabetic rats had a significantly higher PWV compared to the control rats across a supraphysiological range of MAP (170–200 mmHg), but not at a lower MAP range. The non-linear PWV-MAP curve for the diabetic treated group was similar to that of diabetic untreated group, indicating that aortic stiffness was similar at every given level of MAP (Figure 1). Although all three groups received similar doses of phenylephrine, MAP above 170 mmHg was not achieved in diabetic treated rats. The reason for this effect is unknown, but may possibly include a lower sensitivity to phenylephrine.

**Table 2:** Resting anesthetic haemodynamic parameters obtained before the administration of vasoactive substances

Group	SBP (mmHg)	DBP (mmHg)	MAP (mmHg)	PP (mmHg)	HR (beats/min)	PWV (m/s)
Control (n = 10)	140 ± 30	106 ± 27	118 ± 28	34 ± 6	364 ± 96	5.0 ± 0.6
Diabetes (n = 9)	135 ± 15	103 ± 18	114 ± 17	31 ± 4	343 ± 53	5.2 ± 0.3
Diabetes + vitamin D (n = 9)	136 ± 14	104 ± 12	115 ± 12	32 ± 3	348 ± 107	4.9 ± 0.3

SBP, systolic blood pressure; DBP, diastolic blood pressure; MAP, mean arterial pressure; PP, pulse pressure; HR, heart rate; PWV, pulse wave velocity

*p* > 0.05 between all groups.

### Histological analysis and morphometric parameters

**Figure 2:**
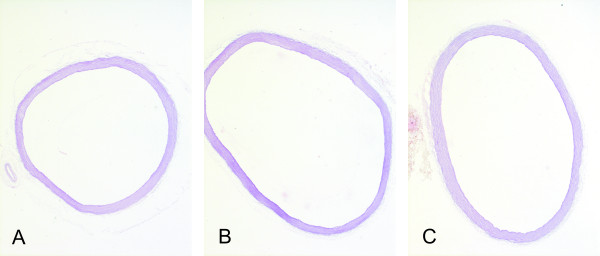
**Micrographs of the transverse aortic sections in the control group (a), untreated diabetic group (b), and diabetes + vitamin D group (c).** Note the enlarged internal diameter of the aorta in the diabetic group (b). Resorcin-fuchsin. Original magnification × 26.

**Figure 3:**
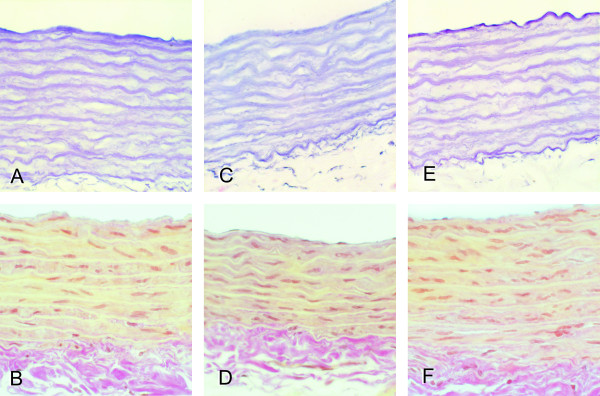
**A panel of micrographs of the aortic sections in the control group (a and b), untreated diabetic group (c and d), and diabetes + vitamin D group (e and f).** Reduction of the thickness of the medial layer (c and d) and disorganisation of elastic lamellae (c) was observed in diabetic untreated rats, while in diabetes + vitamin D group the aortic wall morphology was more similar to the control group samples. Resorcin-fuchsin (a, c, e) and van Gieson (b, d, f). Original magnification × 360.

Aortae of the control group showed a regular vascular morphology, while several alterations were noted in the structure of aortae of rats in the untreated and treated diabetic groups. More pronounced changes were found in the aortae of the untreated diabetic group, where focal irregular arrangement of elastic fibers was noted together with decreased staining intensity of elastic fibers and increased internal diameter of the aorta (Table 3, Figures 2 and 3). Changes in the medial thickness and in collagen staining were not statistically significant compared to the control group (Table 3). Milder changes of the aortic wall, particularly regarding medial elastic fibers with no focal disarrangements were noted in the diabetic treated group (Table 3, Figure 3). Untreated diabetes was also associated with reduced ratio of elastin to collagen that was prevented by vitamin D supplementation (Table 3). Both in untreated and treated diabetic groups no focal thickenings or other significant alterations of the intimal layer were found.

**Table 3:** Morphometric parameters and estimations of staining intensity of connective tissue fibers in the medial and adventitial layers of the thoracic aorta

Parameter	Control (n = 10)	Diabetes (n = 9)	Diabetes + vitamin D (n = 9)
Internal diameter of aorta (mm)	1.40 ± 0.27	1.65 ± 0.18^*^	1.55 ± 0.21
Thickness of media (μm)	86.41 ± 8.15	79.09 ± 14.61	82.93 ± 11.29
Elastic fibers in media (arbitrary units)	2.44 ± 0.33	1.84 ± 0.33^¶;#^	2.29 ± 0.26
Collagen fibers in media (arbitrary units)	1.20 ± 0.38	1.38 ± 0.46	1.24 ± 0.41
Collagen fibers in adventitia (arbitrary units)	2.90 ± 0.41	2.56 ± 0.40	2.52 ± 0.43
Elastin/collagen ratio in media (%)	1.72 ± 0.55	1.33 ± 0.43^*;#^	1.88 ± 0.47

^*^p < 0.05 vs control; ^¶^*p* < 0.01 vs control; ^#^*p* < 0.05 vs diabetes + vitamin D.

## Discussion

In the current study, we have investigated the effects of experimental diabetes on the functional/structural properties of the aorta and the endothelial function and the possible protective effects of vitamin D supplementation. The principal finding of this study was that diabetes was associated with differential effects on the aortic stiffness, demonstrated by higher isobaric PWV at a supraphysiological range of MAP (170–200 mmHg), but not at a lower MAP range. Untreated diabetic rats also exhibited lower levels of serum 25(OH)D and elevated levels of serum ADMA, a marker of endothelial dysfunction. Administration of vitamin D for 10 weeks significantly increased the levels of serum 25(OH)D, but did not protect from aortic stiffening as evidenced by isobaric PWV. Serum ADMA levels were also not affected by vitamin D supplementation. However, vitamin D effectively preserved the structure of elastic fibers and the ratio of elastic fibers to collagen fibers in the aortic media.

STZ-induced diabetes is a well-accepted experimental model of uncontrolled type 1 DM and studies have reported STZ-induced impairment of aortic elastic properties in rats [23,24]. Increased arterial stiffness has also been demonstrated in Zucker diabetic fatty rats, an animal model of type 2 DM. [25,26]. These findings have been supported by clinical observations, which have shown associations between increased aortic PWV and type 1 or type 2 DM [5,27,28]. In accordance with previous studies, we demonstrate increased aortic stiffness in STZ-diabetic rats compared to nondiabetic rats.

It is important to note that as PWV is strongly dependent on BP [29], measurements of PWV can be accurately and independently compared only if obtained over a range of BP that is often not feasible to induce in patients. We assessed PWV over a wide range of MAP using phenylephrine and nitroglycerine to raise and lower MAP, respectively. As a result, we found that differences in isobaric PWV only became evident at high levels of MAP which demonstrates that intrinsic aortic stiffness was not increased in diabetic animals at the physiological BP levels. These results indicate that, during the course of diabetes, early changes in the arterial integrity are reflected in the increased central artery stiffness that can remain undetected since blood pressure may not be elevated in that stage. Increased aortic stiffness can be established when diabetic patients develop hypertension, which highlights the importance of adequate and early blood pressure control for prevention of diabetic macrovascular complications.

The possible mechanisms implicated in the differential effects of diabetes on aortic stiffness may include alterations in the proportions and structural integrity of elastic fibers, collagen fibers, or the extracellular matrix in the vessel wall [30]. Biomechanically, collagen fibers mediate stiffness at higher pressure, while elastin provides support at a lower pressure range [31,32]. In our experiment, a decrease in the elastic lamellae in the aortic media was noted in the untreated diabetic group whilst no clear changes in collagen fibers were detected. Nevertheless, a decrease in elastic fibers and a relative increase of collagen in relation to elastin can result in increased stiffness that may become evident at a high-pressure range as was observed in our experiments. The absence of differences in aortic stiffness at a lower BP range may also be attributed to a compensatory mechanism of preserved smooth muscle activity.

Endothelial dysfunction, characterised by impaired production of nitric oxide (NO), plays an important role in the development of diabetic vascular complications [33]. Several studies have indicated that the impairment of endothelium-dependent vasodilation is an important factor contributing to aortic stiffening [9,34]. Our experiment shows that the serum level of ADMA, a marker of endothelial dysfunction, is significantly elevated in STZ-induced diabetes in parallel with impaired structural and functional properties of the aorta. These findings are consistent with studies showing that endothelial dysfunction occurs early in the course of diabetic vascular complications, as evidenced by functional assessment of the endothelium [35,36] and elevated levels of circulating ADMA [37].

Previous studies have reported that treatment with vitamin D may lower arterial blood pressure [38], improve endothelial function [38], and decrease aortic stiffness [39]. In our study, the isobaric PWV in diabetic animals receiving vitamin D was similar to that of diabetic untreated animals. Furthermore, there were no differences in the serum ADMA concentrations between the diabetic groups. These findings may be attributed to the fact that the levels of blood glucose and HbA1c were unaffected by vitamin D supplementation, suggesting that the possible protective effects of vitamin D against diabetes-induced increase in aortic stiffness and endothelial dysfunction may have been abolished by the persistent high-grade hyperglycemia.

Although vitamin D supplementation could not protect from the early impairment of large artery function, it still had a positive effect on relative preservation of elastic fiber organisation in the medial layer of the aorta. The mechanisms involved in this process remain unknown, but may include the down-regulation of the renin-angiotensin system [40], since angiotensin II is known to stimulate tissue remodeling in the arteries [41] and agents that inhibit this system have been shown to have beneficial effects on the structural properties of the arterial wall [41,42]. Nevertheless, the effects of vitamin D supplementation on the diabetic complications at a molecular level remain to be established. Indeed, our preliminary experiments have shown that vitamin D may affect several oxidative stress parameters in STZ-induced diabetes, including serum total antioxidant capacity and advanced glycation end-products (manuscript in preparation), which requires further investigation.

In summary, the present study demonstrated that in rats, STZ-induced diabetes impairs endothelial function and exerts differential effects on aortic stiffness, characterised by the increased PWV at supraphysiological MAP levels, whereas aortic elasticity was preserved at a lower pressure range. Chronic administration of vitamin D did not have an effect on the diabetes-induced aortic stiffness and endothelial dysfunction, but preserved the proportions and integrity of elastic fibers in the aortic media. Further investigations will provide more information on the effects and role of vitamin D in the macrovascular complications of type 1 diabetes.

## Competing interests

The authors declare that they have no competing interests.

## Authors’ contributions

ES performed the experiments, drafted the manuscript, and performed the statistical analysis. TP participated in designing the study and performing the experiments. ES, PK, JK, MZ, JE conceived of the study, participated in its design and coordination, and helped to draft the manuscript. MB and APA analysed the data, participated in the study design and coordination, and helped to draft the manuscript. MA and AA performed the histological analyses and helped to draft the manuscript. All authors have read and approved the final manuscript.
